# Human Cytomegalovirus Impairs the Function of Plasmacytoid Dendritic Cells in Lymphoid Organs

**DOI:** 10.1371/journal.pone.0003482

**Published:** 2008-10-22

**Authors:** Kerstin Schneider, Ursula Meyer-Koenig, Frank T. Hufert

**Affiliations:** 1 Department of Virology, Institute for Medical Microbiology and Hygiene, University of Freiburg, Freiburg, Germany; 2 Institute of Virology, University Medical Center Goettingen, Goettingen, Germany; New York University School of Medicine, United States of America

## Abstract

Human dendritic cells (DCs) are the main antigen presenting cells (APC) and can be divided into two main populations, myeloid and plasmacytoid DCs (pDCs), the latter being the main producers of Type I Interferon. The vast majority of pDCs can be found in lymphoid organs, where the main pool of all immune cells is located, but a minority of pDCs also circulate in peripheral blood. Human cytomegalovirus (HCMV) employs multiple mechanisms to evade the immune system. In this study, we could show that pDCs obtained from lymphoid organs (tonsils) (tpDCs) and from blood (bpDCs) are different subpopulations in humans. Interestingly, these populations react in opposite manner to HCMV-infection. TpDCs were fully permissive for HCMV. Their IFN-α production and the expression of costimulatory and adhesion molecules were altered after infection. In contrast, in bpDCs HCMV replication was abrogated and the cells were activated with increased IFN-α production and upregulation of MHC class I, costimulatory, and adhesion molecules. HCMV-infection of both, tpDCs and bpDCs, led to a decreased T cell stimulation, probably mediated through a soluble factor produced by HCMV-infected pDCs. We propose that the HCMV-mediated impairment of tpDCs is a newly discovered mechanism selectively targeting the host's major population of pDCs residing in lymphoid organs.

## Introduction

Human cytomegalovirus (HCMV) is a beta-herpesvirus that persistently infects the host and causes extensive morbidity and mortality in neonates and immunocompromised patients, including transplant recipients. HCMV is highly species-specific, but can infect a wide range of cell types including endothelial cells and cells of the hematopoietic system [1]–[4]. The gene expression follows a cascade with immediate early (IE) genes coding for regulatory proteins, early genes (E) coding for e.g. viral polymerases, followed by late (L) gene expression coding for structural viral proteins. Synthesis of viral particles is determined at a post entry level, and it has been shown that cellular and viral factors are responsible for the completion of the viral replication cycle [5]–[7]. In non-permissive cells the replication cycle is usually terminated at the level of IE and/or E gene expression [8].

Dendritic cells (DC) are potent antigen presenting cells essential for the initiation of immune responses through priming of naïve or resting T cells [9]. In humans, two main DC subsets have been described; myeloid DCs, which are CD11c^+^, CD33^+^, CD123^+/−^, and plasmacytoid DCs (pDCs), which are CD11c^−^, CD33^−^, and CD123^++^ [10]. These populations differ not only in their phenotypic marker expression but also in functional properties. Myeloid DCs can be derived from a CD34^+^ hematopoietic progenitor cell and are strategically located in peripheral tissues at the entry site of pathogens [11]. After taking up an antigen, they undergo maturation and activate T cells by direct cell-cell contact and by the secretion of cytokines. Plasmacytoid DCs and myeloid DCs are generated in the bone marrow and circulate in peripheral blood in very low numbers [12]. However the origin of different DC populations is still a matter of debate. Evidence obtained from the mouse system indicates that a common myeloid DC/pDC precursor cell exists, since Del Hoyo et al. demonstrated, that blood-derived Lin^−^CD11c^+^MHC-II^−^ progenitors differentiate into spleen CD8^+^, CD8^−^ DC and pDCs, but not into macrophages, after transfer to irradiated mice [13]. Onai et al. also identified a DC precursor cell in mouse bone marrow that gave rise to myeloid DC and pDCs, but not to other cell lineages *in vitro* [14].

However, the vast majority of pDCs can be found in lymphoid organs such as thymus, bone marrow, spleen, tonsils, and lymph nodes. After contact with antigen, pDCs migrate directly from the peripheral blood to the lymphatic tissue using the high endothelial venules for entry. They are predominantly localized in the T cell zone of the lymph nodes, where they rapidly produce a large amount of type I IFN. Here, they activate the innate and adaptive immune responses [15]. Furthermore, pDCs in tonsils are in close contact with CD8^+^ memory T cells and this colocalization allows memory CD8^+^ T cells to control the pDC response to viruses [16]. In addition, pDCs initiate a productive CD4^+^ T cell response in lymph nodes [17]. Since tpDCs play a key role in the early regulation of innate and adaptive immunity they are excellent targets for virus-mediated immune evasion mechanisms.

Viruses that persist in the host have developed multiple strategies to escape from the attack of the immune system [18], [19]. In particular, HCMV has evolved several mechanisms to modulate the host response and to escape immune control. These include blocking of peptide transporters, down-regulation of MHC, costimulatory and adhesion molecules, expression of MHC class I homologues, impaired T cell activation, and interference with the cytokine and chemokine network [20]–[34]. All these mechanisms are used to subvert the inflammatory response during primary HCMV infection and HCMV reactivation.

In a previous study, we showed that endothelial cell adapted HCMV strains replicate in myeloid DCs and impair their function [28]. Recently, Kvale et al. showed that HCMV activate pDCs isolated from peripheral blood mononuclear cells. They concluded that this may explain why healthy people experience HCMV infection and reactivation asymptomatically [35]. In our study we mainly focus on the major pool of pDCs in the host which resides in the lymphatic tissue (tpDCs) [36]–[38]. They are one of the important key players sitting at the edge of innate and adaptive immunity in a compartment, which is driving the host immune response. We demonstrate that tpDCs and bpDCs are different subpopulations, and that they behave in opposite manner upon HCMV infection.

## Results

### BpDCs and tpDCs are two distinct subpopulations

Dendritic cells play a major role in initiating and regulating the immune response. They share a number of common features, like MHC class II expression in combination with an absence of the lineage-specific markers CD3, CD14, and CD19. However, DCs are a heterogeneous group with variations in their phenotype, morphology, function, and tissue localization [36], [39]. Therefore, we characterized pDCs isolated from tonsils or blood by flow cytometry analysis of several surface molecules directly after isolation (Table 1). Both populations were negative for myeloid markers (CD11c, CD33), CD45RO (LCA), and CD95 (FAS), and positive for CD45RA (LCA), the adhesion molecule CD54, costimulatory molecules (CD80, CD86), the IL-3 receptor (CD123), and antigen presenting molecules (MHC class I and MHC class II). They differ in their expression level of the costimulatory molecule CD40 (tpDC^low^; bpDC^−^), the adhesion molecule CD58 (tpDC^−^; bpDC^+^) and in the expression of NKp44 (tpDC^+^; bpDC^−^). As described by Fuchs et al. bpDCs upregulate NKp44 after co-culture with IL-3 in vitro, and crosslinking of NKp44 leads to inhibition of IFN-α production in response to cytosine-phosphate-guanosine (CpG) oligonucleotides [16].

**Table 1:** Characterization of plasmacytoid DCs.

Cell surface marker	tpDC		bpDC	
	Day 0	Day 5	Day 0	Day 5
CD11c	−	low	−	−
CD33	−	−	−	−
CD40	low	+	−	−
CD45RA	+	+	+	+
CD45RO	−	+	−	−
CD54	low	+	low	+
CD58	−	+	+	+
CD80	+	+	low	−
CD86	low	+	+	+
CD95	−	−	−	−
CD123	++	++	+	+
MHC class I	+	++	+	++
MHC class II	+	++	+	+
NKp44	+	−	−	low/+

Flow cytometry analysis of pDCs was performed directly after isolation (day 0) and at day five after incubation with IL-3.

Survival of pDCs in cell culture is IL-3 dependent [40]. Thus, purified tpDCs and bpDCs were re-characterized at day five of incubation with IL-3 by flow cytometry analysis (Table 1). Both subpopulations were negative for CD33 and CD95 and positive for CD45RA, CD54, CD58, CD86, CD123, MHC class I, and MHC class II. However, they differed in their expression level of the costimulatory molecules CD40 (tpDC^+^; bpDC^−^) and CD80 (tpDC^+^; bpDC^−^), the activation molecule CD45RO (tpDC^+^; bpDC^−^) and in the expression of NKp44 (tpDC^−^; bpDC^+^). Therefore, we concluded that tpDCs and bpDCs are two distinct subpopulations.

### HCMV productively infects tpDCs only

In a previous study, we and others showed that myeloid DCs were permissive for HCMV [28]. We investigated whether endothelial cell-adapted HCMV replicates in tpDCs and bpDCs. We isolated pDCs from tonsils or blood of healthy HCMV seronegative donors with a purity of >90%, and characterized the <10% of contaminating cells obtained from tpDCs and bpDCs. The small amount of contaminating cells obtained from tpDCs contained T cells (45–65%), B cells (9–18%), granulocytes (7–12%) and NK cells (16–20%). In preparations of bpDCs, contaminating cells comprised T cells (24–35%), B cells (10–15%), granulocytes (17–40%) and NK cells (20–35%).

**Figure 1:**
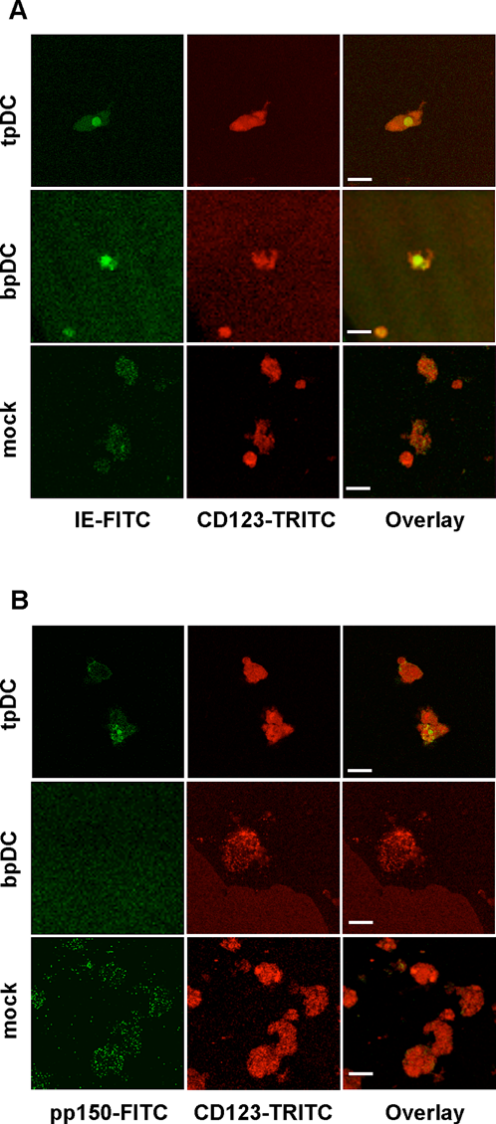
HCMV protein expression in pDCs analyzed by confocal two-colour immunofluorescence microscopy. (A) Expression of HCMV-IE protein (FITC) and CD123 (TRITC) (IL-3R) in HCMV-infected tpDCs and bpDCs and in mock-infected pDCs at day 1 after infection. (B) Expression of HCMV late protein pp150 (FITC) and CD123 (TRITC) (IL-3R) in HCMV-infected tpDCs and bpDCs and in mock-infected pDCs at day five after infection. Bars represent 20 µm.

Productive HCMV infection of tpDCs and bpDCs was investigated by confocal two-colour immunofluorescence microscopy and shell vial culture. Freshly isolated pDCs were either mock-infected or infected with HCMV. Since HCMV gene expression follows a cascade with IE proteins being expressed first, followed by E and L gene expression, pDCs were analyzed for the expression of IE protein at day one and L protein (pp150) at day five post infection using confocal two-colour immunofluorescence microscopy. In figure 1, the co-expression of the pDC marker CD123 (IL-3R) and the viral proteins IE (Fig. 1A) and pp150 (Fig. 1B) are shown. We demonstrated, that IE as well as pp150 proteins were present in HCMV-infected tpDCs only, indicating productive infection. As in non-permissive cells, IE but no late protein (pp150) was present in bpDCs. Staining for viral proteins in mock-infected pDCs yielded negative results. The number of HCMV-infected tpDCs and bpDCs was donor dependent and ranged from 1–10% (tpDCs) to 0.05–1% (bpDCs), respectively (Table 2).

**Table 2:** HCMV-infection rates of plasmacytoid DC.

	HCMV infection rate [%]	
	TpDCs	BpDCs
Donor 1	10	1
Donor 2	5	1
Donor 3	2,5	0,5
Donor 4	1,5	0,05
Donor 5	10	0,1
Donor 6	1	n.d.
Donor 7	5	n.d.
Donor 8	4	n.d.

TpDCs and bpDCs were stained for HCMV IE protein 18 h after infection. Infection rates were calculated as the number of IE positive pDCs compared to IE negative cells. n.d. = not done.

**Figure 2:**
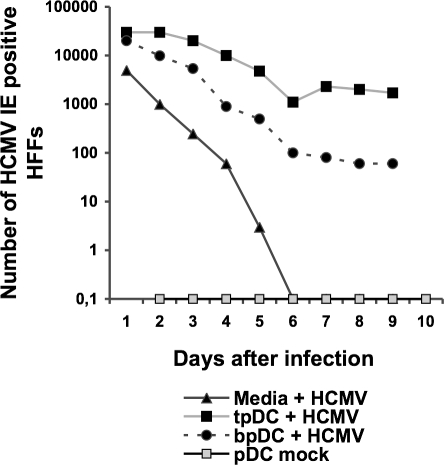
Detection of productive HCMV infection in pDCs. TpDCs and bpDCs were infected with HCMV. Virus stocks were incubated with media only. Supernatant of infected tpDCs and bpDCs and media incubated with HCMV were collected at d1–d9 after infection and added to uninfected HFFs. Virus titers were measured by shell vial culture staining for HCMV IE protein expression in HFFs 18 h post infection.

Productive viral infection was confirmed by shell vial culture and virus production was measured in supernatants obtained from infected tpDCs, bpDCs and from virus stocks incubated with medium only. The latter, to measure virus particle stability under the same culture conditions. Supernatants of mock-infected pDCs served as negative controls. As shown in figure 2, HCMV remained stable in the supernatants up to five days post infection and could not be detected after day six post infection. Both tpDCs and bpDCs infected with HCMV showed a decrease in virus release up to day six post infection. However, after day six post infection the amount of virus slowly decreased in bpDCs, but increased in tpDCs. This indicates a short time interval of HCMV production in tpDCs with only a moderate efficiency. A similar phenomenon was observed by Riegler et al. when myeloid DC were investigated [41].

We conclude that tpDCs but not bpDCs were fully permissive for HCMV since IE and L proteins as well as virus production was found in infected tpDCs only. In contrast, bpDCs were not fully permissive for HCMV, as we only detected IE proteins and failed to detect virus production by shell vial culture.

### HCMV-infection impairs cell surface molecule expression only in tpDCs

**Figure 3:**
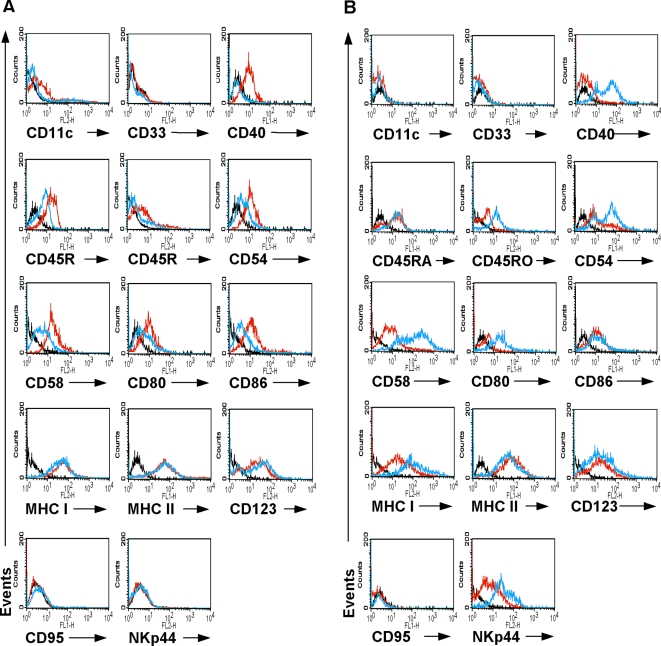
Kinetics of surface marker expression on tpDCs and bpDCs by flow cytometry analysis. HCMV-infected and mock-infected tpDCs (A) and bpDCs (B) were stained with anti-human-CD11c-PE, -CD33-FITC, -CD40-FITC, -CD45RA-FITC, -CD45RO-PE, -CD54-FITC, -CD58-PE, -CD80-FITC, -CD86-PE, -CD95, -CD123-PE, -MHC class I-FITC, -MHC class II-FITC and NKp44-PE at day five after infection. Black lines represent the isotype controls, red lines the marker expression of mock-infected pDCs, and blue lines the marker expression of HCMV-infected pDCs.

HCMV is able to modulate the expression of antigen presenting, adhesion, and costimulatory molecules in myeloid DCs [23], [28], [42]. We analyzed the ability of HCMV to impair the expression of key cell surface molecules on pDCs. These include antigen presenting molecules (MHC class I, MHC class II), costimulatory molecules (CD40, CD80 and CD86), and adhesion molecules (CD11c, CD54, CD58). Furthermore, we analyzed the expression of CD123 (IL-3 receptor), CD95 (FAS), CD33 (myeloid marker), CD45RO/RA (LCA) and NKp44 (activation marker of NK cells). TpDCs and bpDCs were infected with HCMV or left untreated. As a control, UV-inactivated HCMV was incubated with tpDCs or bpDCs. UV-inactivated HCMV is unable to infect cells, but the viral particles are still able to bind to the cell surface and thus act as antigen stimulus. At day six after infection, the cells were stained for CD11c, CD33, CD40, CD45RA, CD45RO, CD54, CD58, CD80, CD86, CD95, CD123, MHC class I, MHC class II, or NKp44 and analyzed by flow cytometry. The results of one representative experiment out of five are shown for tpDCs (Fig. 3A) and for bpDCs (Fig. 3B). Neither mock-infected nor HCMV-infected tpDCs or bpDCs expressed the cell surface molecule CD33 or CD95. In HCMV-infected tpDCs the expression of CD40, and CD45RO was almost completely downregulated, whereas the expression of CD45RA, CD54, CD58, CD80, and CD86 was decreased and the upregulation of CD11c was blocked. No differences in the expression levels of MHC class I, MHC class II, CD123, or NKp44 were observed. In contrast, infected bpDCs upregulated the expression of CD40, CD45RO, CD54, CD58, CD80, MHC class I, and NKp44. The surface molecule CD11c was not expressed on bpDCs either before nor after infection with HCMV. No differences were observed when the expression levels of MHC class II, CD123, CD86, and CD45RA were measured in HCMV-infected or uninfected bpDCs. Furthermore, there was no difference between mock-infected pDCs and pDCs incubated with UV-inactivated HCMV when cell surface molecule expression was analyzed (data not shown).

We conclude that HCMV was able to impair the expression of CD45RA, CD45RO, costimulatory and adhesion molecules on tpDCs but failed to downregulate the expression of antigen presenting molecules. In contrast, HCMV infection led to an activation of bpDCs. This resulted in an upregulation of costimulatory molecules, adhesion molecules, MHC class I and NKp44.

### Infection of tpDCs results in impaired IFN-α production whereas infection of bpDCs leads to an increased secretion

**Figure 4:**
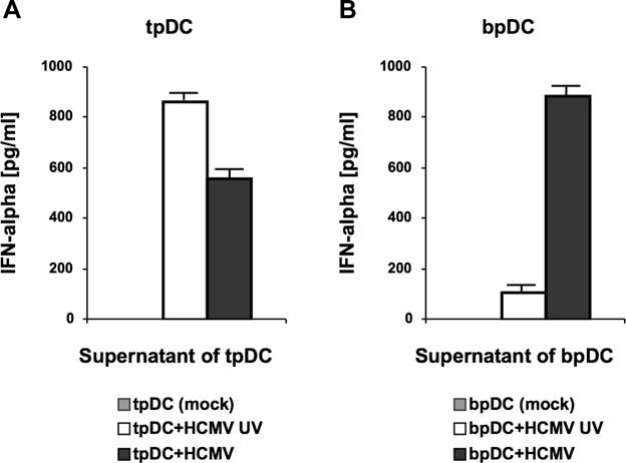
Spontaneous and induced production of IFN-α in tpDCs and bpDCs cultures. Cell culture supernatants from HCMV-infected pDCs (pDC+HCMV), mock-infected pDCs (pDC mock) and from pDCs incubated with UV-inactivated HCMV (pDC+HCMV UV) were collected five days post infection. The IFN-α concentration in the supernatants of tpDCs (A) and bpDCs (B) were measured by ELISA.

The main function of pDCs is the production of the antivirally active cytokine IFN-α. In previous studies, it was observed that HCMV was able to inhibit the production of several cytokines in infected myeloid DCs [28], [31], [34]. Here, we analyzed the capacity of tpDCs and bpDCs to produce IFN-α by ELISA using supernatants of HCMV-infected cells (tpDCs, bpDCs) and cells stimulated with antigen using UV-inactivated virus five days post infection. Mock-infected tpDCs and bpDCs served as negative controls. In figure 4, the mean value of two representative experiments is given. No IFN-α was detected in supernatants of mock-infected cells, whereas antigen stimulation with UV-inactivated HCMV induced cell activation and the production of IFN-α. In this experimental setting the IFN-α production of tpDCs was nine fold higher compared to bpDCs (Fig. 4A and Fig. 4B, white bars), which indicates a higher capacity of tpDC to produce IFN-α upon stimulation. When the IFN-α production of HCMV-infected tpDCs (Fig. 4A, black bar) was analyzed, it turned out that the production of IFN-α was decreased by 10%–40% compared to tpDCs stimulated with UV-inactivated HCMV (Fig. 4A, white bar). The decrease of the IFN-α production correlated with the number of infected cells. A low number of HCMV-infected tpDCs (1–2.5%) cause an IFN-α suppression of up to 10%, whereas a high number of infected tpDCs (4–10%) cause a reduction of the IFN-α production up to 40%. In contrast, in supernatants of HCMV-infected bpDCs (Fig. 4B, black bar) the amount of IFN-α was increased up to 20 fold compared to the IFN-α concentration measured in supernatants of bpDCs incubated with UV-inactivated HCMV (Fig. 4B, white bar). The differences in IFN-α production were statistically significant as measured by chi-square test (p<0.0001).

From these data, we conclude that HCMV selectively impaired the IFN-α production in tpDCs, whereas HCMV induced the production of IFN-α in bpDCs.

### HCMV-infected tpDCs and bpDCs show decreased ability to stimulate T cells

**Figure 5:**
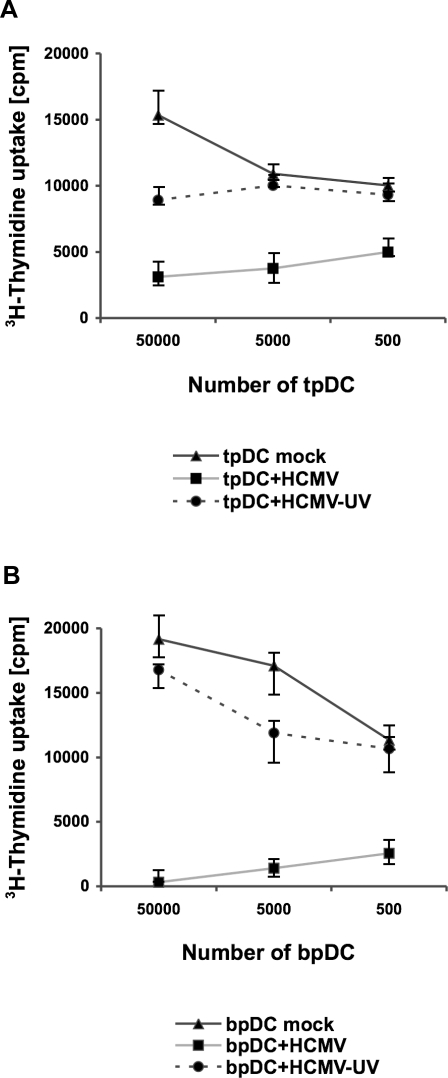
HCMV infection suppressed alloreactivity of pDCs in the MLR. HCMV-infected (MOI 50) pDCs (pDC+HCMV) and pDCs incubated with UV-inactivated virus (pDC+HCMV-UV) were irradiated (30 Gray) at day five post infection. 5×10^4^–5×10^2^ tpDCs (A) or bpDCs (B) were added as stimulator cells to 10^5^ allogeneic purified CD4^+^ T cells (responder cells). As a control, mock-infected tpDCs or bpDCs (pDC mock) were included in each experiment. To measure T cell proliferation, cells were labelled with 1µCi ^3^H-thymidine per well at day five after coculture. The incorporation of ^3^H-thymidine (cpm) was measured for 18 h.

The influence of HCMV-infection on T cell stimulation was investigated by allogeneic mixed lymphocyte reaction (MLR). TpDCs or bpDCs and T cells from a different donor were co-cultivated for five days. In this experimental setting the T cells recognize the foreign MHC molecules and not the antigen presented by the pDCs. As shown in figure 5, the capacity of HCMV-infected tpDCs and bpDCs to induce CD4^+^ T cell proliferation was decreased 3–6 fold for tpDCs and 10–20 fold for bpDCs, when compared to the controls using mock infected cells or UV-inactivated HCMV. One representative result out of five is shown for tpDCs (Fig. 5A) and for bpDCs (Fig. 5B). The ^3^H-thymidine uptakes are expressed as average cpm of triplicate wells. The differences in the ^3^H- thymidine uptakes were statistically significant as measured by chi-square test (p<0.0001).

Similar results were obtained when the non-adherent fraction of PBMC were used instead of CD4^+^ T cells in the MLR (data not shown).

In summary, HCMV-infection of both, tpDCs and bpDCs, led to a decreased allogeneic T cell stimulation. In contrast, UV-inactivated virus did not inhibit lymphocyte proliferation. Therefore, the binding of HCMV to the cell surface of pDCs was not driving this effect.

### A soluble factor is involved in the impairment of the allogeneic MLR

**Figure 6:**
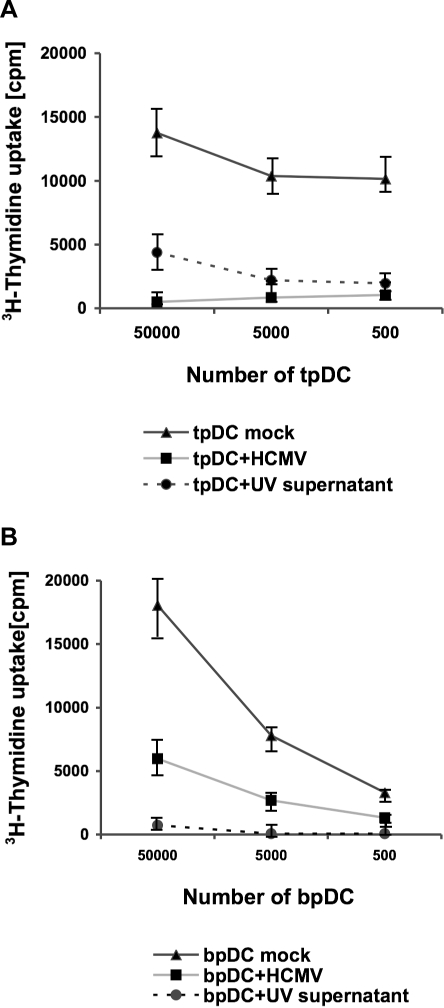
HCMV mediated soluble factor suppressed alloreactivity of pDCs in an MLR. HCMV-infected (MOI 50) pDCs (pDC+HCMV) and pDCs incubated with UV-inactivated supernatant of HCMV infected pDCs (pDC+UV supernantant) were irradiated (30 Gray) at day five post infection. 5×10^4^–5×10^2^ tpDCs (A) or bpDCs (B) were added as stimulator cells to 10^5^ allogeneic purified CD4^+^ T cells (responder cells). As a control, mock-infected tpDCs or bpDCs (pDC mock) were included in each experiment. To measure T cell proliferation, cells were labelled with 1µCi ^3^H-thymidine per well at day five after coculture. The incorporation of ^3^H-thymidine (cpm) was measured for 18 h.

In myeloid DCs, a soluble factor induced by IE and/or E genes has been incriminated in the impairment of T cell activation [28], [32], [43]. Since IE gene products were detected in HCMV-infected tpDCs and bpDCs, we tested the hypothesis that a soluble factor might be involved in the inhibition of the MLR activity. UV-inactivated supernatants were obtained from HCMV-infected tpDCs or bpDCs and transferred to the MLR containing uninfected pDCs and T cells. The transfer of supernatant induced a decrease in CD4^+^ T cell proliferation when tpDCs (3–6 fold) and bpDCs (10–20 fold) were used. The inhibition of the MLR using infected cells or UV-inactivated supernatant transfer yielded similar results. One representative result out of five is shown for tpDCs (Fig. 6A) and for bpDCs (Fig. 6B). The ^3^H-thymidine uptakes are expressed as average cpm of triplicate wells. The differences in the ^3^H- thymidine uptakes were statistically significant as measured by chi-square test (p<0.0001).

From these results we conclude that a soluble factor produced by HCMV-infected tpDCs and bpDCs must be involved in the inhibition of the allogeneic T cell proliferation.

## Discussion

Cytomegaloviruses, which persist lifelong in the host, induce a severe but transient immunosuppression. HCMV-mediated immunosuppression can take weeks to months to resolve and has a critical effect on host survival especially in immunocompromized subjects [1]. In organ and bone marrow transplant recipients opportunistic infections may lead to a more severe clinical outcome as a consequence of HCMV infection. Therefore, it is crucial to understand the mechanisms involved in HCMV-mediated immunosuppression. HCMV has evolved several mechanisms to modulate the host response and to escape immune control [20]–[34]. However, the early impairment of APC such as DCs might play a key role, because the inhibition of the first steps of the host response supports the viral strategy to disseminate and build up latency in the host. Plasmacytoid DCs are the main producer cells of IFN-α and are thought to link innate and adaptive immunity. Especially cells such as pDCs, which are one of the key regulators of both innate and adaptive immunity, are excellent targets, because the impairment of their function simultaneously hampers both pathways of the immune system. The host response is even more severely affected when the main pool of these cells located in the compartments, which are driving the host immune response, are impaired. To follow this line of arguments we have investigated pDCs with a focus on pDCs obtained from lymphatic tissue, where the vast majority of these cells can be found in the host [15], [44]. However, the minority of pDCs circulating in peripheral blood was also investigated.

First, we characterized pDC populations obtained from peripheral blood and lymphatic tissue by flow cytometry analysis of various cell surface markers. It turned out that tpDCs and bpDCs differed in their expression level of costimulatory molecules CD40, CD80 (tpDCs^+^; bpDCs^−^), adhesion molecule CD58 (tpDCs^+^; bpDCs^−^), activation molecule CD45RO (tpDCs^+^; bpDCs^−^), and NKp44 (tpDCs^−^; bpDCs^+^). Since we found different cell surface marker expression patterns of bpDCs and tpDCs directly after isolation and after coculture with IL-3, which activated the cells and started maturation, we speculate that both cell populations are closely related but distinct subpopulations. Microarray analysis data of Lindsted et al. showed that human bpDCs and tpDCs are very closely related based on the expression patterns of chemokine receptor, IL receptors, and pattern recognition receptors. The correlation determined by Lindsted et al. using hierarchical clustering between tpDCs and bpDCs was 0.91 [45]. The phenotype analysis of Linsted et al. was indeed different to our analysis, because we investigated in part other cell surface molecules such as CD33, CD54, CD58, CD95, MHC class I, and NKp44. Thus, our results are not entirely contradictory.

It is quite obvious that bpDCs and tpDCs are close relatives and one may speculate that as for many hematopoietic cells the blood borne bpDCs may be the precursor cells of tpDCs located in the lymphoid tissues since DCs in lymphoid tissues are in a dynamic balance, with an estimated half-life of only a few days during steady-state conditions [46]. This turnover mandates a continuous replacement of DCs by a precursor population. In the mouse system there is much evidence that a common precursor cell of myeloid DC/pDC exists. Del Hoyo et al. demonstrated, that blood-derived Lin^−^CD11c^+^MHC-II^−^ progenitors differentiate into spleen CD8^+^, CD8^−^ DCs and pDCs, but not into macrophages, after transfer to irradiated mice [13]. Onai et al. identified also a DC precursor in mouse bone marrow that gave rise to myeloid DC and pDCs, but not to other cell lineages *in vitro* and Naik et al. identified a clonal DC precursor that closely resembles the common DC precursor [14], [47]. It may be fair to speculate that as for many haematopeitic cells the blood borne bpDCs might be the precursor cells of tpDCs located in the lymphoid tissues and that bpDCs can differentiate into tpDCs after migrating to the lymphoid organs.

However, the main goal of our investigations was to study the interaction of pDCs with HCMV with a main focus on pDCs obtained from lymphatic tissue. We looked at HCMV replication in pDCs and whether the function of these cells was altered as a consequence of infection. To show viral replication in pDCs, we applied confocal two-colour immunofluorescence microscopy and measured virus production by shell vial culture. The complete viral replication cycle was detected only in tpDCs, whereas in bpDCs the replication cycle was abrogated before L proteins were expressed. Low-level viral replication was determined by shell vial culture in tpDCs only. In contrast to endothelial cells, which replicate HCMV very efficiently and are known as the main site of viral replication in the host, tpDCs are only low-level virus producers. As shown here, a very efficient virus production in tpDCs was not a prerequisite to switch off their function since the main biological aim, which is to counteract the first steps of the host immune response, is easily achieved by low-level virus replication in tpDCs.

In addition, it turned out that in bpDC the virus replication cycle abrogated before L proteins were expressed. The difference of bpDCs and tpDCs to support HCMV replication can be explained by the stage of cell differentiation and the presence of certain cellular factors as shown by an in vitro model of the monocyte/macrophage system. Here, the presence of certain transcription factors drove productive HCMV infection; a lack of these factors resulted in a HCMV restricted gene expression of the IE genes [6], [48]. In addition, we and others have previously shown that HCMV can infect CD34^+^ progenitor cells in the bone marrow, but this infection was not productive. These progenitor cells serve as a reservoir of latent virus with limited transcription of viral genes. However, infectious virus could be recovered only from terminally differentiated cells. The difference between bpDCs and tpDCs in HCMV replication further supports the hypothesis that these cells belong to different subpopulations [5], [6], [8], [49].

After we had shown that HCMV can infect bpDCs and tpDCs but only replicates in tpDCs we investigated whether HCMV impairs pDC-function. Here, we investigated the cell surface molecule expression, the production of IFN-α, and the capacity to stimulate T cells.

It is well known that cytomegaloviruses employ multiple mechanisms to circumvent the host response. One mechanism is to interfere with the MHC class I pathway in a multistep manner. As a result of HCMV infection myeloid DCs downregulate the expression of MHC- class I, which impairs antigen presentation [28]. Surprisingly, MHC class I molecule expression in HCMV-infected tpDCs and bpDCs was not altered. The molecular mechanism responsible for this difference still remains to be elucidated. However, there was a clear difference in the marker profile expression when HCMV-infected bpDCs and tpDCs were compared. BpDCs were simply activated which resulted in an increased expression of all key cell surface molecules such as antigen presenting molecules, adhesion molecules and costimulatory molecules. In HCMV-infected tpDCs however, a downregulation of adhesion and costimulatory molecules was observed, whereas no impairment of antigen presenting molecules occured. This might not be due to the fact that only up to 10% of these cells are infected because a HCMV-mediated downregulation of adhesion and costimulatory molecules occurred in all cells analyzed by flow cytometry. Thus, HCMV-mediated downregulation of cell surface markers does not occur in infected cells only. This represents first evidence, that, as described for myeloid DCs, a soluble factor may be involved [28], [32], [43]. The decrease of key molecules on tpDCs is the first evidence that the function of the main pool of pDCs, and thus the lymphatic tissue of the host, is impaired as a consequence of HCMV infection.

The main function of pDCs is the production of IFN-α and it is well known that in response to certain viruses, like cytomegaloviruses, pDCs produce high amounts of IFN-α [50], [51], which is important for the first line of antiviral host response. The disruption of IFN-α signalling in IFNR-αβ knockout mice, for example, rendered them highly susceptible to mouse cytomegalovirus infection [52]. In the study presented here, HCMV infection of bpDCs resulted in stimulation of IFN-α production compared to uninfected controls. These data are in accordance with Kvale et al. and Varani et al., who focussed exclusively on bpDCs and myeloid DCs from peripheral blood [35], [53]. Interestingly, the capacity of the IFN-α production after stimulation with antigen was nine fold lower in bpDCs compared to tpDCs. This also supports the hypothesis that bpDCs could be precursor cells of tpDCs, since immature precursor cells might not have yet gained full functional activity and fail to produce high amounts of IFN-α. On the other hand HCMV-infection impaired the production of IFN-α in tpDCs, but failed to suppress IFN-α production in bpDCs. A possible explanation is that this effect strictly depends on the number of cells infected. Small number of infected tpDCs (1–2.5%) led to a reduction of only 10% whereas a high number of infected tpDCs (up to 10%) induced a decrease of IFN-α production up to 40%. Since the numbers of infected bpDCs is up to 20fold lower compared to tpDCs no suppression of the IFN-α production was observed. In summary, the production of IFN-α was altered in tpDCs but not in bpDCs as a consequence of HCMV infection in vitro. This might have an impact on the function of the lymphatic tissue during HCMV infection. However, the situation in vivo remains to be elucidated.

Another key function of DCs is the induction of a T cell response. Thus, we investigated this issue using pDCs from blood and tonsils. Our data are in accordance with Varani et al. who could show that HCMV hamper the allostimulatory ability of bpDCs [53]. In addition, we could show an impairment of the MLR in tpDCs. This indicates that in HCMV infection T cell stimulation might be impaired in the lymphatic tissue. Since T cell stimulation of both, bpDCs and tpDCs, was affected the expression of IE genes was sufficient to achieve this effect. It is known that myeloid DCs are more powerful inducers of a T cell response compared to pDCs. Thus, the proliferation rate using pDCs was lower as previously observed for myeloid DCs [28], [54]. However, the transfer of UV-inactivated supernatants obtained from HCMV-infected pDCs to uninfected pDCs prior to allogeneic T cell stimulation also caused a severe impairment of the MLR. This clearly shows that, as in myeloid DCs, a soluble inhibitory factor was involved in the T cell impairment [28], [32], [43]. It could be argued that cross-presentation of DCs might be a mechanism to bypass HCMV-induced immunosuppression, since early NK cell killing of HCMV-infected cells can induce antigen presentation very early on in non-infected DCs [55]. A countermeasure to the effect of cross-presentation is the production of the soluble factor which induces inhibition of T cell proliferation in pDCs as shown here and as described previously for myeloid DC [28], [32], [43]. Furthermore, Benedict et al. have shown very recently that murine cytomegalovirus programs infected DCs during acute infection to inhibit the T cell response of the host by tipping the balance between negative and positive cosignals [56]. The relevance of the different steps in the impairment of HCMV-mediated T cell function still remains to be defined.

In summary, we could show that based on the expression of cell surface markers human pDCs obtained from peripheral blood and from lymphatic tissue are two distinct subpopulations, which also differ in their capability to permit replication and production of HCMV. TpDCs could be productively infected with HCMV and this led to a severe impairment of cell function including a reduced IFN-α production and a downregulation of costimulatory, adhesion, and activation molecules. In contrast, the abrogated replication of HCMV in bpDCs resulted in cell activation with an upregulation of several surface molecules and a stimulation of IFN-α production. Interestingly, in both populations an HCMV-mediated soluble factor was involved in the impairment of T cell stimulation. The almost selective impairment of pDC function in lymphoid organs, targets the main pool of these cells in the compartment where the host generates the immune response. This is a new immune evasion mechanism employed by HCMV.

## Materials and Methods

### Viruses

Clinical HCMV strains were kindly provided from Ch. Sinzger (Institut fuer medizinische Virologie, Tuebingen, Germany). The following endothelial cell adapted virus stocks were used: AA07/E, KSA16/3, TB40/E, TB42/E. Virus stocks were grown on endothelial cells and generated by ultracentrifugation as described previously [28].

### Preparation of plasmacytoid DCs

TpDCs were isolated from tonsils of healthy, HCMV seronegative humans. This was approved by the ethic committee of the university hospital of Freiburg (for details refer to: http://www.uniklinik-freiburg.de/ethik-kommission/live/index.html) and tissue samples were only investigated at the university hospital of Freiburg. Informed verbal and written consent was obtained from all patients. Tonsillectomy was performed at the department of otorhinolaryngology head and neck surgery, university hospital Freiburg, Germany. Fresh tonsils were cut into small fragments and added to RPMI 1640 (CCpro, Neustadt an der Weinstrasse, Germany) supplemented with 1 mg/ml Collagenase and 0.25 mg/ml DNAse (Roche Diagnostics, Wiesbaden, Germany). After incubation (40 min, 37°C) the suspension was pushed through a nylon mesh (70 µm), resuspended in PBS containing 2% FCS and separated by Ficoll gradient (Pharmacia, Upsala, Schweden). For enrichment of tpDCs the interphase cells were collected and purified using the depletion step of the Blood Dendritic Cell Isolation Kit I (Miltenyi Biotec, Bergisch Gladbach, Germany) followed by the BDCA-4 Cell Isolation Kit (Miltenyi Biotec, Bergisch Gladbach, Germany).

BpDCs were isolated from buffy coats of healthy, HCMV seronegative donors. Buffy coats were obtained commercially from the department of transfusion medicine, university hospital Freiburg, Germany. The use of buffy coats for research purposes was approved by the ethics committee of the university hospital of Freiburg (http://www.uniklinik-freiburg.de/ethik-kommission/live/index.html). Prior blood donation informed written consent was obtained. Peripheral blood mononuclear cells were isolated by Ficoll gradient (Pharmacia, Upsalla, Sweden). For enrichment of bpDCs, the interphase cells were collected and purified using the same protocol as for tpDCs. TpDCs and bpDCs were placed in culture using RPMI 1640, 10% FCS and 2 mM glutamine supplemented with IL-3 (10 ng/ml, R+D Systems, Wiesbaden, Germany).

### Infection of plasmacytoid DCs

For all experiments pDCs were infected with an MOI of 50 from a pool of equal amounts of different purified HCMV strains. The pDCs were incubated for 24 h with infectious supernatant, washed, and placed in culture.

HCMV infection rates were measured by shell vial culture as described previously [28] using immunoflourescence staining for HCMV IE protein expression 18 h post infection on HFF.

To calculate HCMV virus titers in infected pDCs, supernatants of HCMV infected tpDCs and bpDCs were collected between day one and day nine after infection. These supernatants were diluted between 10 fold to 10^6^ fold in tissue culture medium and added to uninfected HFFs. Total amount of HCMV IE positive HFFs were counted 18 hours post infection. The virus titers were then calculated by the dilution factor and the number of HCMV IE positive cells.

### Confocal immunofluorescence microscopy

To perform a two-colour immunofluorescence staining, pDCs were harvested at day one (detection of HCMV IE protein) and at day five after infection (detection of HCMV pp150 protein). The cells were first incubated with CD123-Biotin (BD Pharmigen, Heidelberg, Germany) followed by Streptavidin-Alexa546 (Molecular probes, Leiden, Netherlands). The cells were fixed and permeabilized in acetone/methanol. After incubation with anti-HCMV-IE (Biosoft,Varilhes, France) or anti-HCMV-pp150 (MAK CMV pxp1 a kind gift from Dr. Walter, Behring, Marburg, Germany), an anti-mouse-IgG1-FITC antibody was used to detect IE and pp150 expression (BD Pharmingen, Heidelberg, Germany). The slides were analysed with a confocal microscope (DM IRBE, Leica, Wetzlar, Germany).

### Flow cytometry analysis

Infected and mock-infected pDCs were collected at day five after infection, washed and incubated with one or two of the following monoclonal Antibodies: anti-CD11c-PE (S-HCL-3, BD Pharmingen, Heidelberg, Germany), anti-CD33-FITC (D3HL60-251, Dianova, Hamburg, Germany), anti-CD40-FITC (5C3, BD Pharmingen), anti-CD45-RA-FITC (HI100, BD Pharmingen), anti-CD45-RO-PE (UCHL1, BD Pharmingen), anti-CD54-FITC (84H10, Immunotech, Marseille, France), anti-CD58-PE (AICD58, Immunotech), anti-CD80-FITC (BB1, BD Pharmingen), anti-CD86-PE (IT2.2, BD Pharmingen), anti-CD95-FITC (DX2, BD Pharmingen) anti-CD123-PE (9F5, BD Pharmingen), anti-MHC class I-FITC (G46-2.6, BD Pharmingen), anti-MHC class II-FITC (Tü39, BD Pharmingen), anti-NKp44-PE (Z231, Beckman Coulter, Marseille, France). The samples were analyzed on a FACSsort™ (Becton Dickinson, Heidelberg, Germany) using the CellQuest Pro™ software.

### Cytokine detection

Production of IFN-α was measured in culture supernatants using commercially available ELISA-kits (human IFN-α Module Set, Bender MedSystems, Vienna, Austria) according to the manufacturers recommendations. Supernatants of mock-infected, HCMV-infected and with UV-inactivated HCMV strains incubated pDCs were measured. Cytokines were determined at day six after infection.

### Allogeneic mixed lymphocyte reaction

HCMV-infected (MOI 50) and mock-infected pDCs were irradiated (30 Gray) at day five post infection. Different numbers of pDCs (5×10^4^–5×10^2^) were added to 10^5^ allogeneic purified CD4^+^ T cells (isolated with the CD4^+^ T cell isolation kit (Miltenyi Biotec, Bergisch Gladbach, Germany)) or PBL per well. To measure T cell proliferation, cells were labelled with 1µCi ^3^H-thymidine (Pharmacia, Uppsala, Sweden) per well at day five after coculture, incubated for 18 h, harvested on filters and counted with a Trace-96β-Counter (Berthold, Wildbad, Germany). Each experiment was done in triplicate and was performed using infected, mock-infected and UV-light inactivated virus stocks. In the transfer experiment settings, 50 µl of UV-inactivated supernatant obtained from uninfected or HCMV-infected pDCs was added.

### Statistical analysis

A chi-square test was applied to analyze differences in IFN-α-production and ^3^H-thymidine uptakes by contingency tables using GraphPad Prism 4.0.

## References

[1] Naniche D, Oldstone MB (2000). Generalized immunosuppression: how viruses undermine the immune response.. Cell Mol Life Sci.

[2] Sinzger C, Jahn G (1996). Human cytomegalovirus cell tropism and pathogenesis.. Intervirology.

[3] von Laer D, Meyer-Koenig U, Serr A, Finke J, Kanz L (1995). Detection of cytomegalovirus DNA in CD34+ cells from blood and bone marrow.. Blood.

[4] von Laer D, Serr A, Meyer-König U, Kirste G, Hufert FT (1995). Human cytomegalovirus immediate early and late transcripts are expressed in all major leukocyte populations in vivo.. Journal of Infectious Diseases.

[5] Sinclair J, Sissons P (1996). Latent and persistent infections of monocytes and macrophages.. Intervirology.

[6] Liu R, Baillie J, Sissons JG, Sinclair JH (1994). The transcription factor YY1 binds to negative regulatory elements in the human cytomegalovirus major immediate early enhancer/promoter and mediates repression in non-permissive cells.. Nucleic Acids Res.

[7] Brune W, Menard C, Heesemann J, Koszinowski UH (2001). A ribonucleotide reductase homolog of cytomegalovirus and endothelial cell tropism.. Science.

[8] Ellsmore V, Reid GG, Stow ND (2003). Detection of human cytomegalovirus DNA replication in non-permissive Vero and 293 cells.. J Gen Virol.

[9] Avigan D (1999). Dendritic cells: development, function and potential use for cancer immunotherapy.. Blood Rev.

[10] Penna G, Sozzani S, Adorini L (2001). Cutting edge: selective usage of chemokine receptors by plasmacytoid dendritic cells.. J Immunol.

[11] Larsson M, Beignon AS, Bhardwaj N (2004). DC-virus interplay: a double edged sword.. Semin Immunol.

[12] Liu YJ (2005). IPC: professional type 1 interferon-producing cells and plasmacytoid dendritic cell precursors.. Annu Rev Immunol.

[13] del Hoyo GM, Martin P, Vargas HH, Ruiz S, Arias CF (2002). Characterization of a common precursor population for dendritic cells.. Nature.

[14] Onai N, Obata-Onai A, Schmid MA, Ohteki T, Jarrossay D (2007). Identification of clonogenic common Flt3+M-CSFR+ plasmacytoid and conventional dendritic cell progenitors in mouse bone marrow.. Nat Immunol.

[15] Yoneyama H, Matsuno K, Zhang Y, Nishiwaki T, Kitabatake M (2004). Evidence for recruitment of plasmacytoid dendritic cell precursors to inflamed lymph nodes through high endothelial venules.. Int Immunol.

[16] Fuchs A, Cella M, Kondo T, Colonna M (2005). Paradoxic inhibition of human natural interferon-producing cells by the activating receptor NKp44.. Blood.

[17] Sapoznikov A, Fischer JA, Zaft T, Krauthgamer R, Dzionek A (2007). Organ-dependent in vivo priming of naive CD4+,but not CD8+,T cells by plasmacytoid dendritic cells.. J Exp Med.

[18] Hengel H, Koszinowski UH (1997). Interference with antigen processing by viruses.. Curr Opin Immunol.

[19] Wiertz EJ, Mukherjee S, Ploegh HL (1997). Viruses use stealth technology to escape from the host immune system.. Molecular Medicine Today.

[20] Chee MS, Satchwell SC, Preddie E, Weston KM, Barrell BG (1990). Human cytomegalovirus encodes three G protein-coupled receptor homologues.. Nature.

[21] Wiertz EJ, Jones TR, Sun L, Bogyo M, Geuze HJ (1996). The human cytomegalovirus US11 gene product dislocates MHC class I heavy chains from the endoplasmic reticulum to the cytosol.. Cell.

[22] Jones TR, Wiertz EJ, Sun L, Fish KN, Nelson JA (1996). Human cytomegalovirus US3 impairs transport and maturation of major histocompatibility complex class I heavy chains.. Proc Natl Acad Sci U S A.

[23] Ahn K, Gruhler A, Galocha B, Jones TR, Wiertz EJ (1997). The ER-luminal domain of the HCMV glycoprotein US6 inhibits peptide translocation by TAP.. Immunity.

[24] Browne H, Smith G, Beck S, Minson T (1990). A complex between the MHC class I homologue encoded by human cytomegalovirus and beta 2 microglobulin.. Nature.

[25] Streblow DN, Soderberg-Naucler C, Vieira J, Smith P, Wakabayashi E (1999). The human cytomegalovirus chemokine receptor US28 mediates vascular smooth muscle cell migration.. Cell.

[26] Michelson S, Alcami J, Kim SJ, Danielpour D, Bachelerie F (1994). Human cytomegalovirus infection induces transcription and secretion of transforming growth factor beta 1.. J Virol.

[27] Alcami J, Barzu T, Michelson S (1991). Induction of an endothelial cell growth factor by human cytomegalovirus infection of fibroblasts.. J Gen Virol.

[28] Beck K, Meyer-Konig U, Weidmann M, Nern C, Hufert FT (2003). Human cytomegalovirus impairs dendritic cell function: a novel mechanism of human cytomegalovirus immune escape.. Eur J Immunol.

[29] Raftery MJ, Schwab M, Eibert SM, Samstag Y, Walczak H (2001). Targeting the function of mature dendritic cells by human cytomegalovirus: a multilayered viral defense strategy.. Immunity.

[30] Raftery MJ, Wieland D, Gronewald S, Kraus AA, Giese T (2004). Shaping phenotype, function, and survival of dendritic cells by cytomegalovirus-encoded IL-10.. J Immunol.

[31] Moutaftsi M, Mehl AM, Borysiewicz LK, Tabi Z (2002). Human cytomegalovirus inhibits maturation and impairs function of monocyte-derived dendritic cells.. Blood.

[32] Senechal B, Boruchov AM, Reagan JL, Hart DN, Young JW (2004). Infection of mature monocyte-derived dendritic cells with human cytomegalovirus inhibits stimulation of T-cell proliferation via the release of soluble CD83.. Blood.

[33] Grigoleit U, Riegler S, Einsele H, Laib Sampaio K, Jahn G (2002). Human cytomegalovirus induces a direct inhibitory effect on antigen presentation by monocyte-derived immature dendritic cells.. Br J Haematol.

[34] Andrews DM, Andoniou CE, Granucci F, Ricciardi-Castagnoli P, Degli-Esposti MA (2001). Infection of dendritic cells by murine cytomegalovirus induces functional paralysis.. Nat Immunol.

[35] Kvale EO, Dalgaard J, Lund-Johansen F, Rollag H, Farkas L (2006). CD11c+ dendritic cells and plasmacytoid DCs are activated by human cytomegalovirus and retain efficient T cell-stimulatory capability upon infection.. Blood.

[36] Summers KL, Hock BD, McKenzie JL, Hart DN (2001). Phenotypic characterization of five dendritic cell subsets in human tonsils.. Am J Pathol.

[37] Briere F, Bendriss-Vermare N, Delale T, Burg S, Corbet C (2002). Origin and filiation of human plasmacytoid dendritic cells.. Hum Immunol.

[38] McKenna K, Beignon AS, Bhardwaj N (2005). Plasmacytoid dendritic cells: linking innate and adaptive immunity.. J Virol.

[39] Hill S, Coates JP, Kimber I, Knight SC (1994). Differential function of dendritic cells isolated from blood and lymph nodes.. Immunology.

[40] Grouard G, Rissoan MC, Filgueira L, Durand I, Banchereau J (1997). The enigmatic plasmacytoid T cells develop into dendritic cells with interleukin (IL)-3 and CD40-ligand.. J Exp Med.

[41] Riegler S, Hebart H, Einsele H, Brossart P, Jahn G (2000). Monocyte-derived dendritic cells are permissive to the complete replicative cycle of human cytomegalovirus.. J Gen Virol.

[42] Miller DM, Rahill BM, Boss JM, Lairmore MD, Durbin JE (1998). Human cytomegalovirus inhibits major histocompatibility complex class II expression by disruption of the Jak/Stat pathway.. J Exp Med.

[43] Chang WL, Baumgarth N, Yu D, Barry PA (2004). Human cytomegalovirus-encoded interleukin-10 homolog inhibits maturation of dendritic cells and alters their functionality.. J Virol.

[44] Zhang Z, Wang FS (2005). Plasmacytoid dendritic cells act as the most competent cell type in linking antiviral innate and adaptive immune responses.. Cell Mol Immunol.

[45] Lindstedt M, Lundberg K, Borrebaeck CA (2005). Gene family clustering identifies functionally associated subsets of human in vivo blood and tonsillar dendritic cells.. J Immunol.

[46] Liu K, Waskow C, Liu X, Yao K, Hoh J (2007). Origin of dendritic cells in peripheral lymphoid organs of mice.. Nat Immunol.

[47] Naik SH, Sathe P, Park HY, Metcalf D, Proietto AI (2007). Development of plasmacytoid and conventional dendritic cell subtypes from single precursor cells derived in vitro and in vivo.. Nat Immunol.

[48] Sinclair JH, Baillie J, Bryant LA, Taylor-Wiedeman JA, Sissons JG (1992). Repression of human cytomegalovirus major immediate early gene expression in a monocytic cell line.. J Gen Virol.

[49] Soderbergnaucler C, Fish KN, Nelson JA (1997). Reactivation of latent human cytomegalovirus by allogeneic stimulation of blood cells from healthy donors.. Cell.

[50] Asselin-Paturel C, Boonstra A, Dalod M, Durand I, Yessaad N (2001). Mouse type I IFN-producing cells are immature APCs with plasmacytoid morphology.. Nat Immunol.

[51] Dalod M, Salazar-Mather TP, Malmgaard L, Lewis C, Asselin-Paturel C (2002). Interferon alpha/beta and interleukin 12 responses to viral infections: pathways regulating dendritic cell cytokine expression in vivo.. J Exp Med.

[52] Presti RM, Pollock JL, Dal Canto AJ, O'Guin AK, Virgin HWt (1998). Interferon gamma regulates acute and latent murine cytomegalovirus infection and chronic disease of the great vessels.. J Exp Med.

[53] Varani S, Cederarv M, Feld S, Tammik C, Frascaroli G (2007). Human cytomegalovirus differentially controls B cell and T cell responses through effects on plasmacytoid dendritic cells.. J Immunol.

[54] Summers KL, Hock BD, McKenzie JL, Hart DN (2001). Phenotypic characterization of five dendritic cell subsets in human tonsils.. Am J Pathol.

[55] Arrode G, Davrinche C (2003). Dendritic cells and HCMV cross-presentation.. Curr Top Microbiol Immunol.

[56] Benedict CA, Loewendorf A, Garcia Z, Blazar BR, Janssen EM (2008). Dendritic cell programming by cytomegalovirus stunts naive T cell responses via the PD-L1/PD-1 pathway.. J Immunol.

